# Entomological Investigations, Seasonal Fluctuations and Impact of Bioclimate Factors of Phlebotomines Sand Flies (Diptera: Psychodidae) of an Emerging Focus of Cutaneous Leishmaniasis in Aichoun, Central Morocco

**DOI:** 10.1155/2020/6495108

**Published:** 2020-07-07

**Authors:** Fatima Zahra Talbi, Abdelhakim El Ouali Lalami, Mouhcine Fadil, Mohamed Najy, Hassan Ech-Chafay, Mohamed Lachhab, Said Lotfi, Nordine Nouayti, Khadija Lahouiti, Chafika Faraj, Abdellatif Janati Idrissi

**Affiliations:** ^1^Laboratory Biotechnology and Preservation of Natural Resources, Faculty of Sciences Dhar El Mahraz, Sidi Mohamed Ben Abdellah University, 30000 Fez, Morocco; ^2^Laboratory of Medical Entomology, National Institute of Hygiene, 27 Avenue Ibn Battuta, Agdal, 11400 Rabat, Morocco; ^3^Higher Institute of Nursing Professions and Health Techniques of Fez, Regional Health Directorate Fez-Meknes, EL Ghassani Hospital, 30000 Fez, Morocco; ^4^Physio-Chemical laboratory of Inorganic Materials, Materials Science Center (MSC), Ecole Normale Supérieure, Mohammed V University in Rabat, Morocco; ^5^Laboratory of Agro-Physiology, Biotechnology, Environment and Quality, Department of Biology, University Ibn Tofail, Faculty of Science, BP133, 14000 Kenitra, Morocco; ^6^Applied Sciences Laboratory, Water and Environmental Engineering Team, National School of Applied Sciences, Al Hoceima. Abdelmalek Essaadi University, Morocco; ^7^Laboratory of Microbial Biotechnology, Department of Biology, Faculty of Sciences and Technology, University Sidi Mohamed Ben Abdellah, BP 2202, Road of Immouzer, Fez, Morocco

## Abstract

Leishmaniasis diseases are endemic in Morocco. An entomological survey was conducted in Aichoun locality for 1 year from September 2013 to August 2014. The objective of this study was to investigate the sand fly fauna, mainly the species composition and the monthly species prevalence in accordance with bioclimate factors. Sand flies were collected twice a month, using sticky traps and CDC light traps. During a one-year study, 4472 specimens of sand fly were caught (72.56% male/22.44% female) that were composed of seven species divided into two genera: *Phlebotomus* (99.46%) and *Sergentomyia* (0.53%). *Ph. sergenti* was the most prevalent species (46.64%), followed by *Ph. perniciosus* (38.19%), *Ph. longicuspis* (9.32%), *Ph. papatasi* (5.23%), and *Ph. ariasi* (0.06%). The genus *Sergentomyia* was even less frequent. The population dynamics showed a bimodal trend with two peaks: the first one in October (12.03% of specimens) and the second in June (27.92% of specimens). The study of the effects of climatic factors in the study area showed a link between the dynamics of sand flies and the variation of these parameters (temperature and relative humidity). During the period between November and March, the sand flies were absent. The highest prevalence of sand flies was recorded in June when the temperature and relative humidity values reached, respectively, 25.8°C and 42%. The minimum number of specimens was collected in September with an average temperature of 23.19°C and relative humidity of 57.4%. Statistical analysis with principal component analysis has shown a strong positive correlation between temperature parameters and the seasonal distribution of sand flies. The climatic factor of relative humidity has been judged of being negatively correlated. The wind speed does not have any impact on the relative abundance of all species. Within this context, the results will be useful for the development of a monitoring program to better manage the operations and evaluate their effectiveness.

## 1. Introduction

Both zoonotic visceral leishmaniasis (ZVL) and cutaneous leishmaniasis (CL) are endemic in Morocco [1]. The incidence of CL (cutaneous leishmaniasis) peaked up in 2010 with 2263 cases and then remained stable in 2011 and 2012 with 2100 and 2137 cases, respectively [2]. The causative agents are *Leishmania major*, which is transmitted by *Phlebotomus* (*Phlebotomus*) *papatasi* (Scopoli); *Leishmaniatropica*, transmitted by *Phlebotomus* (*Paraphlebotomus*) *sergenti* Parrot [3]; and *L. infantum* which is transmitted by *Ph. ariasi*; *Ph. perniciosus* and *Ph. longicuspis* are the usual vectors [4, 5]. Between 2004 and 2013, the total number of 24 804 cases of *L. major*CL and 16 852 cases of *L. tropica* CL were recorded in Morocco. For the cases of *L. infantum* CL, a few epidemiological data are available. They are represented with a few sporadic cases in the north of the country [6].

It is known that different biotic and abiotical variables may affect the seasonality of phlebotomine sand flies. The impact of climate, urbanization, proximity of humans and domestic animals, organic matter in the soil, and vegetation type has a significant role in the distribution and abundance of sand fly populations [7]. However, there is no information on the relationship between environmental and climatic conditions and seasonality of different sand flies in this region.

Thus, the continuous survey of vectors and their control is required by health services in order to prevent the spread of leishmaniasis epidemic. The present paper gives results of a one-year-long study of the seasonal fluctuations undergone by the common sand flies species populations in Aichoun locality, a focus of leishmaniasis in central Morocco, which were analyzed in relation to the meteorological variations.

The working hypothesis was that data on the environmental and meteorological factors affecting the density and distribution of both vectors could provide a clear idea about the spatial and temporal trends.

## 2. Material and Methods

### 2.1. Study Area

**Figure 1:**
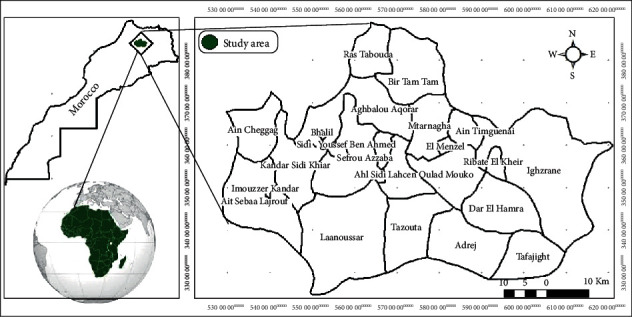
Presentation of Province Sefrou, Commune of Tazouta (Aichoun locality).

The study was conducted in a rural locality (Aichoun) situated in Sefrou Province, in the northwest of the Moroccan Middle Atlas (Figure 1). The study area is characterized by a semiarid climate, hot and dry in the summer, cold and rainy in the winter, with a temperature ranging from 2 to 40°C, and total annual rainfall about 400 mm while the mean altitude is around 750 m.

### 2.2. Sand Fly Sampling

Sand fly captures were carried out in a period from September 2013 to August 2014. Nine collecting sites were chosen in Aichoun locality. Collections were gathered by using sticky papers (21 × 27.3 cm) coated with castor oil. We used 64 traps in each trapping campaign. The compound was sampled for two nights each month with papers placed at fixed interior stations for a 12 h dusk to-dawn period.

**Figure 2:**
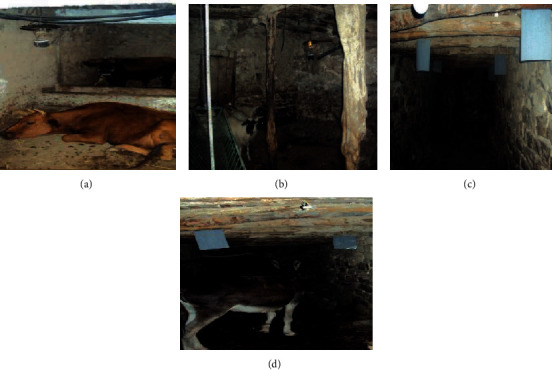
Images of some animal shelters where CDC miniature light-traps (image a-b) and sticky papers (image c-d) were set in Aichoun locality.

Sand flies were also caught by CDC miniature light traps, set out in five stations. During each month of survey, ten CDC light traps were placed at low level (0.5–0.7 m from the ground) and were operated from sunset to sunrise. Sand fly counts from light traps were averaged to yield the mean number of sand flies/trap night (Figure 2).

### 2.3. Morphological Identification of Phlebotomine Species

Captured phlebotomine specimens were stored at 70% of ethanol. Specimens were identified morphologically using keys adapted from Lewis (1978) and Killick-Kendrick et al. 1991 [8, 9]. Female identification was based on the shape of spermathecae and the disposition of the teeth in the pharyngeal armature, whereas males were identified based on their genitalia (hypopygium) [10]. The differentiation between males of *Ph. perniciosus* and *Ph. longicuspis* was made by examining both the copulatory valves shape form and the number of coxite hairs [11, 12].

### 2.4. Recording Climatic Conditions

To determine whether the local variations might affect abundance or activity of sand flies in the study area, we placed two data loggers. They were programmed to record temperature and humidity in the study area during the whole year. Every month, we retrieved these parameters from the data logger and obtained the recorded averages.

### 2.5. Data Analysis

Various parameters and ecological indexes were determined for the data analysis [13, 14].

- Relative abundance. (RA)% = *n*/*N* × 100 (*n*: number of specimens of species *x*; *N*: total number of specimens in the sample).
- Frequency of occurrence (C). Ci = ri × 100/*R* (ri: is the total number of samples containing the species considered, *R*: the total number of specimens taken).
- Sex ratio. SR = *F*/*M* × 100 (*F*: number of females; *M*: number of males)
- Specific richness (S). Number of species in a given area.
- Shannon-Wiener index (Hs). Hs = 3.322 [Log *N* − 1/*N* (*Σ*ni Log ni)].
- *N*. absolute abundance; ni: the number of individuals of each species.
- Equitability. *E* = Hs′/*H*′max × 100. (Hs′: the specific diversity observed; *H*′max: the logarithm of the total number of species (*S*) in the sample) *H* (Max) = maximal diversity = 3.322 Log *S*.

### 2.6. Statistical Analyses

Statistical analyses were performed by using the principal component analysis (PCA) to investigate the existence of a correlation between the abundance of the sand flies, average monthly temperature, and average monthly relative humidity in the study area. Analyses were performed by using the Unscrambler software (version 9.7).

## 3. Result

### 3.1. Entomological Survey

During the one-year study, 4472 sand fly specimens were caught, 72.56% of which were males. The specific richness, abundance, density, and Shannon-Weiner index (Hs′) of captured sand flies are shown in Table 1. Seven species belonging to the genera *Phlebotomus* (99.46%) and *Sergentomyia* (0.53%) were identified. *Ph. sergenti* was the most prevalent species (46.64%), followed by *Ph. perniciosus* (38.19%), *Ph. longicuspis* (9.32%), *Ph. papatasi* (5.23%), and *Ph. ariasi* (0.06%). The genus *Sergentomyia* was less frequent. The predominant species was *S. minuta* which represented 0.44% of the total collected sand flies, followed by *S. fallax* 0.08%. The sex ratio was in favour of males for all species with the exception of *S. fallax*. In both types of trap collection, male sand flies were predominant (72.56%) compared to female ones.

Adhesive traps are proving fairly effective in catching sand flies. But, the majority of collected sand flies specimens were captured by CDC for this location during this study period. This amounts to the fact that the CDC traps are always arranged in sheltered and secure places; on the other hand, those sand flies are insect photophilous.

*Ph. sergenti*, *Ph. perniciosus*, and *Ph. papatasi* are the most commune species; they are the most occurrences species in space and thus exhibit the largest spectrum of distribution. In contrary, the rest of the species vary between rare and accessory species in the space (lower frequency of occurrence than 50%). Commune species are those making higher constancy of values or equal to 50% and, therefore, have omnipresence in space. There was a difference in the diversity of the sand fly fauna in Aichoun locality, as indicated by the values of Shannon-Weiner index (Hs′).

The ratio of Shannon diversity and the maximum diversity has given a value greater than 0.5, so the population of sand flies tends towards a balance. The study area is heterogeneous enough to contain species with different requirements and meet them.

### 3.2. Seasonal Survey

**Figure 3:**
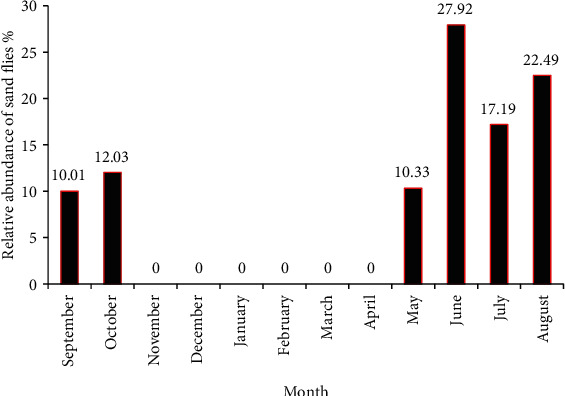
Mean monthly relative abundance of sand flies' fauna in the study area, surveyed from September 2013 to August 2014.

The evolution of the mean monthly relative abundance of the total fauna during the study period is showed in Figure 3. As this figure shows, sand flies were dynamic from May to October in this semiarid area. Fluctuations in abundance of sand flies were observed from September 2013 to August 2014. It was marked by two periods: the first one corresponds to September-October with a peak in October (12.03% of specimens) and the second one, very importantly corresponds to May-June-July-August with a peak in June (27.92% of specimens).

**Figure 4:**
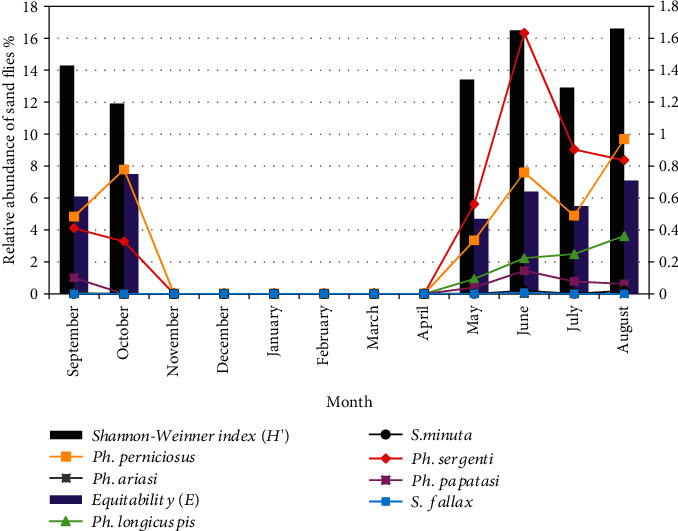
Monthly distribution of sand flies, Shannon-Weinner index (*H*′), Equitability (E) (2013-2014).

The seasonal trends shown by both sand flies' species are illustrated in Figure 4. The greatest value of species richness is noted in June with 7 species. This particular index is void of November to April. Two evolution models were distinguished. *Ph. sergenti* and *Ph. longicuspis* presented a monophasic model. They were active from May to October for *Ph. sergenti* and to September for *Ph. longicuspis* while they were absent in the rest of the year. *Ph. perniciosus* and *Ph. papatasi* displayed a biphasic trend with a first, more important peak in August for the first one (9.98%) and in June for the second vector (1.45%). The other remaining species are present with low density.

In June, the highest number of specimens was marked with the presence of all seven species collected during the study period, followed by May, August, and September with a group of five specimens, while July and October are, respectively, marked by richness equal to four and three. However, during August, the diversity of sand flies in this locality was very high (Hs′ = 1.66; *E* = 0.71) with the four species of *Ph. sergenti*, *Ph. perniciosus*, Ph*. longicuspis*, and *Ph. papatasi* and in October with (Hs′ = 1.19; *E* = 0.75), yet there were three specimens among the five potential vector species of leishmaniasis in Morocco (*Ph. sergenti*, *Ph. perniciosus*, and *Ph. papatasi*). The months of August and October are considered the satisfactory periods to ensure a well-balanced biotope for the most abundant sand flies in the locality of Aichoun.

#### 3.2.1. Effect of Climate Conditions

**Figure 5:**
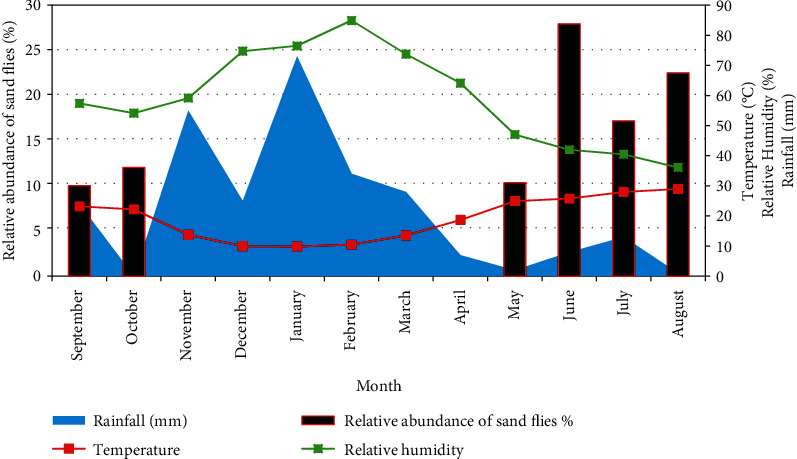
Representation of relative abundance of sand flies and variation of the monthly temperature and relative humidity in Aichoun locality, from September 2013 to August 2014.

With regard to climate, in the periods of high activity, the maximum and minimum monthly temperature ranged between 25.99 and 30.34°C, and 23.9 and 27.67°C, respectively. In June, when the maximum of sand flies (1249) was collected, the average temperature and relative humidity were found to be 25.8°C and 42%, respectively, while the minimum number of sand flies was sampled in September (448) with an average temperature of 23.19°C and 57.4% of relative humidity (Figure 5). From November to April, the seasonal activity of sand flies is null since it is the rainy season.

#### 3.2.2. Statistical Analysis

Data were analyzed using Unscrambler software (version 9.7). The significance difference of the effect of climate conditions was analyzed in relation to sand flies species caught in locality of Aichoun using principal component analysis (PCA).

For data processing by PCA, we used twelve variables from which seven species of sand flies were studied in Aichoun locality. In order to determine the number of components to remember, we will adopt the standard of Kaiser. At a normalized PCA, we retain the components where in the eigenvalues are greater than 1. By referring to this criterion, it is shown that the three first components are sufficient to explain all of the data.  Table 2 shows the number components and the initial values that show the contribution of each of the components to the total variance.

The examination of the numerical results of the PCA shows that the first component explains 62.722% of the data variability while the second one explains 14.896%. So, we can be satisfied to retain these two components explaining 77.618% of total data variability.

**Figure 6:**
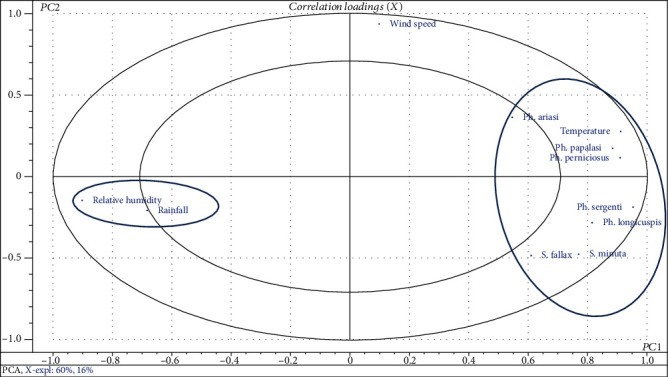
Representation of variables on the factorial plane F1 and F2.

To facilitate the visualization of point clouds, they were thrown into a two-dimensional space. The percentage of inertia explained by the two axes forming a plane is 77.618% of the total variance. These two axes are considered to describe the distribution of variables and individuals on the main level.  Figure 6 reveals the projection of variables in the space of factorial axes F1 and F2.

The correlation circle (Figure 6) shows that eight of twelve variables taken into account in the PCA contribute to the definition of factorial plan F1 × F2. The positive structuring variable of F1 is *Ph. sergenti*, *Ph. longicuspis*, *Ph. papatasi*, *Ph. perniciosus*, *Ph. ariasi*, *S. minuta*, *S. fallax*, and temperature factor. Thus, the axis F1 can be compared to an axis reflecting the parameters favorable to the proliferation of sand flies. Principal components analysis showed that temperature parameter is strongly correlated with the distribution of the seven species of sand flies caught in the locality of Aichoun. On the other hand, relative humidity and precipitation do not have a positive effect on the existence and abundance of these seven species; the correlation was marked strongly negative. However, they are positively correlated with one another. The wind speed setting is considered an independent factor.

In order to confirm the observed correlations between the climate conditions influenced the behavior and the development of sand flies, we have an option to use correlation tests (Table 3). Thus, it is seen that only the temperature factor and the diversity of the seven species of sand flies are positively correlated.

**Figure 7:**
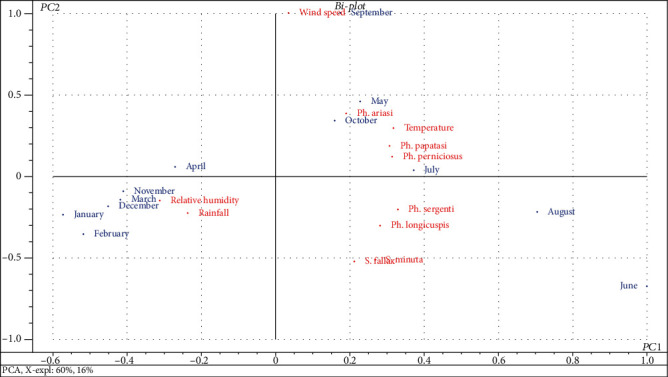
Representation of the variables on the factorial planes F1 and F2.

Examining the factorial plan has allowed to visualize the positive correlation. The Biplot graph looks like both ecological parameters and their correlations with the seasonal distribution of all the sand flies collected during 2014 (Figure 7). The distribution of the month throughout the year 2014 is characterized by two groups. High temperatures are noticed during the months of May, June, July, August, September, and October. It has a positive impact on the relative abundance of sand flies. The five species incriminated in the transmission of leishmaniasis in Morocco have a common behavior towards temperature. The positive correlation is justified by the significant correlation index. The other parameters (relative humidity and precipitation) are noticed with a high value; the species shall enter into diapause period.

These are limiting factors to certain values for the biological development and distribution of all the sand flies collected in the study area. The wind speed has no effect on the relative abundance of all species.

## 4. Discussion

Among 23 species described in Morocco, seven sand fly species (30.43%) were identified in this work. The identified species belong to two genera: *Ph*. *lebotomus* sand *Sergentomyia*. The species of the first genus belong to three subgenera: *Ph*. *lebotomus*, *Paraphlebotomus*, and *Larroussius*.

In *Paraphlebotomus* subgenus, we identified one species: *Ph. sergenti*, the proven vector of *L. tropica* in Morocco [3]. *Ph. sergenti* is very abundant in all the territory of Morocco [15, 16]. But its wide distribution is localized in zones with arid and subhumid climate [17]. According to Boussa et al. [18], this specimen is highly responsive in urban areas with an altitude varying up to 1400 m; this result has also been proved by other authors [7, 19]. Depending on the nature of the soil, *Ph. sergenti* prefers habitats with a sandy texture [20].

At locality of Aichoun during 2013 and 2014, *Ph. sergenti* presents a single-phase seasonal evolution with a single remarkable peak in June (16.32%). This species is marked by a different distribution characterized by the absence of a second peak recorded in 2012 [21]. This result could be explained by the effect of climate. In September, the precipitation of the area during the study period was 24 mm and the wind speed (7.2 m/s) was high compared to the other months. These meteorological settings could influence the distribution of this species.

In *Larroussius* subgenus, we reported three species: *Ph. perniciosus*, *Ph. longicuspis* and *Ph. ariasi*. All of these species are proven vectors of L. infantum [22]. They are very common in Morocco. *Ph. longicuspis* is collected in all altitudes but with a density between 600 and 799 m altitude [23]. However, *Ph. perniciosus* was found in areas at an altitude of 800 to 1000 m and occupies sand soils [24].

For *Ph. perniciosus*, the monthly change showed a biphasic activity with a high prevalence in the month of August (9.68%). This result is consistent with that obtained in the province of Chichaoua [23]. In 2012, this species had a second peak in September [21]. However, in this study, we observed a second peak displacement towards October 2014, which would also have been linked to the ecological factors of the zone (precipitation with 0 mm and wind speed with 3.68 m/s).

*Ph. longicuspis* was reported with 3.62%, in which a high density was revealed in August. The latter asserts what Guernaoui et al. found in the province of Chichaoua [25]. According to some authors, the distribution is favored by local environmental factors (precipitation and temperature), biotic factors (abundance and dynamics of vertebrate hosts), and physical factors (habitat availability) [26, 27]. Morocco is characterized by a great diversity of climate and ecology; altitude alone does not appear to be influencing the distribution of sand flies, but climatic factors would play a role, they could explain the dynamics observed in different species, which were caught during the study period. At the level of our study area, the sand fly fauna activity only appears in the period from May to October, which corresponds to the warm season.

Seasonal studies at Aichoun locality, during a period of December to March, show that sand flies were absent. This result parallels what Alten et al. [28] wrote; apparently, no risk for leishmaniasis transmission took place from December through March in the years considered.

Ph*. papatasi* was the only species that belongs to *Ph*. *lebotomus* genus in our locality. It is known as vector of *L. major* in Morocco [22]. This species suspected to be a vector of zoonotic cutaneous leishmaniasis disease [29]. It is present with two peaks during the period from May to October. This is also the case at the Marrakech level by Boussa et al. [30]. *Ph. papatasi* has been well adapted with arid climatic conditions [29, 30]. It is known for its broad distribution in the Mediterranean basin [31] and according to some studies, these species are considered a plain species because its abundance is remarkable in the sites at an altitude varying from 400 and 600 m and its absence between 1200 and 1400 m [23]. Even if these species are classified among the sand flies present in Aichoun locality, the recorded cases of Cutaneous form are from *L. tropica* and not from *L. major*. Indeed, no declaration was made at the level of the Fez-Boulemane region.

The distribution of sand fly depends on local environmental factors, such as precipitation and temperature, biotic factors, such as the dynamic and abundance of vertebrate hosts and physical factors (geographical barriers and habitat availability) [26, 27]. Morocco is characterized by a large climatic and ecologic diversity; the altitude alone does not seem to be a selective factor for sand fly distribution. Bioclimate and many factors have a great role; mainly, climatic factors seem to better explain dynamic sand fly in Morocco.

In Aichoun locality, the activity of phlebotomine fauna appears half year from May to October which corresponds to the dry season when the weather gets dry. Sand flies were active especially during the period from May to October. Two activity peaks were noted in June and August. In these months, the precipitation rate (8 mm; 1 mm) and the wind speed (1.46 m/s, 3.01 m/s) were low. The specific richness was maximal in June; it was marked by seven species. The optimum humidity for the proliferation of sand flies' hovers around 42%. As such, the density decreased when the humidity exceeded 42% from September to April.

The maximum of sand flies was collected in June, the average temperature found to be 25.8°C; while the minimum number of sand flies was sampled in September with an average temperature of 23.19°C. In the same region Fez Boulmane, especially in the Moulay Yaâcoub province, the temperature and the humidity are the main factors for proliferation of the disease. Indeed, in Ouled Aid, a focus of leishmaniasis, the sand fly density reaches its maximum when the temperature is between 30 and 35°C and disappears when the temperature exceeds 35°C [32]. In mountainous areas near Marrakech with an arid climate, the period of sand flies activity was from June to November with a monophasic evolution for all species presented [33]. Boussaa et al. shows that the sand flies were active in the whole year especially in the period from May to November [30].

In this present study, the statistical analysis by PCA showed another issue, the temperature factor has a considerable effect on activity of sand flies. The correlation between these specimens and the temperature variation is strongly positive. It has a role in their seasonal distribution.

Temperature has a significant effect on phlebotomineactivity, at a certain time. It may act as a limiting factor with low value on the abundance of sand flies [34]. We noted that low temperature values and very warm periods are a limiting factor to the sand flies' activity.

The relative humidity of the air was found to be significantly negatively correlated with the density of sand flies. Indeed, the maximum of the species and the high monthly prevalence marked in the month of June are accompanied with a relative humidity of 42%. The minimum is retained with a relative humidity of 57.4%. But outside the interval (36.1%-57.4%), the sand flies enter in the diapause phase. For other ecological settings (wind speed, precipitation), no significant correlation was found with density. The potential vector species of the leishmaniasis diseases in Aichoun locality were presented with a very high prevalence in high temperature range. These comparative studies could be explained by an adaptation of the different species of sand flies with the climate type.

## 5. Conclusion

To conclude, this study enables us to understand the existing relationship between the density, the seasonal distribution of phlebotomine fauna, and the meteorological factors in Aichoun locality, a focus of leishmaniasis in central Morocco. The activity of sand flies varies between May and October, which corresponds to the periods of risk in this area. The obtained results will be useful for developing a control program, to direct operations and to evaluate the effectiveness in order to prevent the leishmaniasis risks.

## Figures and Tables

**Table 1:** Percentage, the Shannon-Weiner diversity index (Hs′), richness, and frequency of occurrence of the collected sand flies during the study period at Aichoun locality.

Genus	Subgenus	Species	Total collected with sticky traps		Sex ratio	Total collected with CDC		Sex ratio	FO		Hs′	*E*
			*M*	*F*		*M*	*F*		C (%)	Scale		
*Phlebotomus*	*Larroussius*	*Ph. longicuspis*	8	19	0.42	339	51	6.64	25	A	1.66	0.60
		*Ph. perniciosus*	143	26	5.5	915	624	1.46	50	C		
		*Ph. ariasi*	0	0	—	1	2	0.5	25	A		
	*Paraphlebotomus*	*Ph. sergenti*	1386	122	11.36	302	276	1.09	50	C		
	*Phlebotomus*	*Ph. papatasi*	99	27	3.66	41	67	0.61	50	C		
*Sergentomyia*		*S. minuta*	0	3	—	11	6	1.83	16.66	R		
		*S. fallax*	0	0	—	0	4	—	8.33	VR		

*F*: female; *M*: male; FO: Frequency of occurrence; Hs′: Shannon–Weiner index; A: Accessory; C: Commun; R: Rare; VR: Very rare.

**Table 2:** Contribution of number components of the total variance under gone by PCA analysis.

Number component	Initial values		
	Eigenvalues	% of variance	% cumulative
1	7.52665	62.722	62.722
2	1.78757	14.896	77.618
3	1.28481	10.707	88.325

**Table 3:** Representation of important results of the PCA in variables (correlations among variables).

	T(°C)	Rh (%)	Prec (mm)	WS (m/s)	PS	PPe	PL	PP	PA	SM	SF
T° (°C)	1										
RH (%)	-0.9468	1									
Prec (mm)	-0.7471	0.6247	1								
WS (m/s)	0.3047	-0.1971	-0.1041	1							
PS	0.797	-0.8092	-0.5277	-0.0807	1						
PPe	0.8557	-0.8276	-0.6514	0.2059	0.7854	1					
PL	0.7647	-0.8039	-0.478	-0.1821	0.8016	0.7329	1				
PP	0.7769	-0.7243	-0.5432	0.2781	0.8563	0.859	0.5244	1			
PA	0.4567	-0.3924	-0.2956	0.4434	0.5574	0.3409	0.1379	0.6043	1		
SM	0.5305	-0.5904	-0.3691	-0.2714	0.7618	0.6926	0.7876	0.5452	0.2582	1	
SF	0.2885	-0.3397	-0.2005	-0.3479	0.7556	0.378	0.3593	0.6214	0.5222	0.6742	1

T: temperature; HR: relative humidity; Prec: precipitation; WS: wind speed; PS: *Ph. sergenti*; PPe: *Ph. perniciosus*; PL: *Ph. longicuspis*; PP: *Ph. papatasi*; PA: *Ph. ariasi*; SM: *S. minuta*; SF: *S. fallax*.

## Data Availability

No data were used to support this study.
